# Treatment Persistence and Discontinuation with Rivaroxaban, Dabigatran, and Warfarin for Stroke Prevention in Patients with Non-Valvular Atrial Fibrillation in the United States

**DOI:** 10.1371/journal.pone.0157769

**Published:** 2016-06-21

**Authors:** Craig I. Coleman, Muralikrishna Tangirala, Thomas Evers

**Affiliations:** 1 University of Connecticut School of Pharmacy, Storrs, Connecticut, United States of America; 2 Bayer HealthCare Pharmaceuticals, Whippany, New Jersey, United States of America; 3 Bayer Pharma AG, Wuppertal, Germany; University of Bologna, ITALY

## Abstract

A retrospective cohort analysis of the US MarketScan claims databases was performed to compare persistence and discontinuation rates between the vitamin K antagonist warfarin and the non-vitamin K antagonist oral anticoagulants rivaroxaban and dabigatran in patients with non-valvular atrial fibrillation. The analysis included adult patients with non-valvular atrial fibrillation newly initiated on rivaroxaban, dabigatran, or warfarin between November 1, 2011 and December 31, 2013, with a baseline CHA_2_DS_2_-VASc score ≥2, two or more non-valvular atrial fibrillation diagnosis codes (427.31), and ≥6 months’ continuous medical and pharmacy benefit enrollment before oral anticoagulant initiation. Propensity score matching was performed to match patients receiving rivaroxaban with those receiving dabigatran (1:1) and warfarin (1:1). Patients were followed until the first event of inpatient death, end of continuous enrollment, or end of study period. Medication persistence was defined as absence of a refill gap of >60 days. Discontinuation was defined as no additional refill for >90 days and through to end of follow-up. Hazard ratios (HRs) of oral anticoagulant persistence and discontinuation were estimated using Cox proportional hazard models. In total, 3,2634 patients were included (n = 10878/oral anticoagulant group). Rivaroxaban was associated with better persistence than both dabigatran (HR 0.64, 95% confidence interval [CI] 0.62–0.67) and warfarin (HR 0.62, 95% CI 0.59–0.64) and lower discontinuation than dabigatran (HR 0.61, 95% CI 0.58–0.64) and warfarin (HR 0.65, 95% CI 0.62–0.68). Real-world analysis of oral anticoagulant use may reveal whether the relatively high persistence/low discontinuation demonstrated for rivaroxaban translates into lower rates of stroke.

## Introduction

Optimal and persistent use of non-vitamin K antagonist (VKA) oral anticoagulants (OACs) is essential in reducing the risk of ischemic stroke in patients with non-valvular atrial fibrillation (NVAF) [1,2]. Numerous studies have found that persistence rates for VKA therapy for stroke prevention are suboptimal, and are lower than those for non-VKA OACs [3–8]. Dabigatran and rivaroxaban have been used in routine clinical practice in the United States since their approvals in October 2010 and November 2011, respectively [9,10]. However, to date, no single study has compared the persistence and discontinuation rates of rivaroxaban, dabigatran, and the VKA warfarin. Therefore, the objective of this analysis was to compare persistence and discontinuation rates between warfarin and the non-VKA OACs rivaroxaban and dabigatran in patients with NVAF using a large US administrative claims database.

## Methods

This analysis utilized data from the US Truven Health MarketScan administrative claims database [11]. MarketScan combines two separate databases, the Commercial Claims and Encounters and the Medicare Supplemental and Coordination of Benefits databases, to cover all age groups. The databases contain claims from approximately 100 employers, health plans, and government and public organizations, representing about 30 million covered lives in the United States. MarketScan captures health plan enrollment records; participant demographics; International Classification of Diseases, Ninth Revision, Clinical Modification (ICD-9-CM) diagnosis codes; admission and discharge dates; inpatient mortality data; outpatient medical services data; and outpatient prescription drug dispensing records. All data included in the MarketScan database are anonymized and are in compliance with the Health Insurance Portability and Accountability Act of 1996 (HIPAA) to preserve participant anonymity and confidentiality. Because this study used only anonymized patient records and did not involve the collection, use, or transmittal of individually identifiable data, Institutional Review Board approval was not required.

Using a retrospective matched-cohort design, rates of persistence and discontinuation were compared among patients with NVAF prescribed warfarin, rivaroxaban, or dabigatran. For the current analysis, patient data were collected between May 1, 2011 and March 31, 2014. The start date was selected based on the latest approval date of these agents (in this case, rivaroxaban) for stroke prevention in patients with NVAF. The index period, in which OAC-naïve patients were newly initiated on rivaroxaban, dabigatran, or warfarin (index date), was between November 1, 2011 and December 31, 2013. Again, this index period was selected based on the latest approval date among the study agents (in this case, rivaroxaban) for stroke prevention in patients with NVAF. The same index period was used to ensure a baseline period of at least 6 months of continuous medical and prescription benefits coverage. It further ensured an equal chance of therapy switching for all three OACs. Patients were followed until the first event out of inpatient death, the end of continuous enrollment, or the end of the study period.

This analysis included adult patients (age ≥18 years at index date) with a baseline CHA_2_DS_2_-VASc score ≥2 [1], two or more ICD-9-CM diagnosis codes for atrial fibrillation (427.31) at any time during the study period, and ≥6 months of continuous medical and prescription benefits coverage before the index date (i.e. baseline period). We excluded pregnant women, patients taking multiple anticoagulants of interest on the index drug day, and those with transient (ICD-9-CM: 415.x, 429.4; CPT-4: 33400–33999) or valvular (ICD-9-CM: 394.x-397.x, 424.x, 746.0x- 746.7x, V42.2, V42.3; CPT-4: 33400–33478) atrial fibrillation or malignant cancers (ICD-9-CM: 140.x-208.xx, 230.x-234.x) during the 6-month pre-index period.

Propensity score matching was performed to minimize the presence of baseline differences between the OAC cohorts. Patients were matched in a 2-phase process using a “greedy” matching procedure [12]. The initial phase matched patients receiving rivaroxaban with those receiving dabigatran, and the second phase matched selected rivaroxaban patients with those receiving warfarin. Patients were matched according to their baseline demographics: age (categorical), gender, region, insurance plan type, Charlson comorbidity index score [13], and individual risk stratification scores of CHADS_2_/CHA_2_DS_2_-VASc [1], ATRIA [14], and HAS-BLED [15]. Comorbidities overlapped between the different scores and included congestive heart failure, hypertension, diabetes, ischemic stroke/transient ischemic attack, vascular disease, anemia, prior major bleeding events, abnormal renal function, abnormal liver function, and excess alcohol consumption [1,14,15].

The primary outcome of this study was medication persistence, which was measured during the follow-up period. Medication persistence with OAC therapy was defined as the absence of a refill gap of >60 days (non-persistence was defined as a gap of >60 days after the end of the day’s supply of the medication of interest). Discontinuation was defined as no additional refill of the medication of interest for >90 days through to the end of follow-up. For this reason, only patients with ≥90 days’ enrollment after discontinuation of the medication were assessed for this latter outcome.

Categorical variables were summarized as percentages, and continuous variables were summarized using means ± standard deviations. Differences in baseline characteristics between the rivaroxaban-, dabigatran-, and warfarin-treated cohorts were assessed by calculating standardized differences. Baseline characteristics with standardized differences of <10% were considered sufficiently balanced [16]. Kaplan–Meier curves were generated to depict the time to non-persistence and discontinuation over the follow-up period. Cox proportional hazard regression models were estimated based on the matched cohorts to examine the impact of OAC choice on time to non-persistence and discontinuation. No additional independent variables were included in the Cox proportional hazards models. For all models, hazard ratios (HRs) and 95% confidence intervals (CIs) were reported. Data management and statistical analysis were performed using SAS version 9.4 (Cary, North Carolina).

## Results

**Fig 1:**
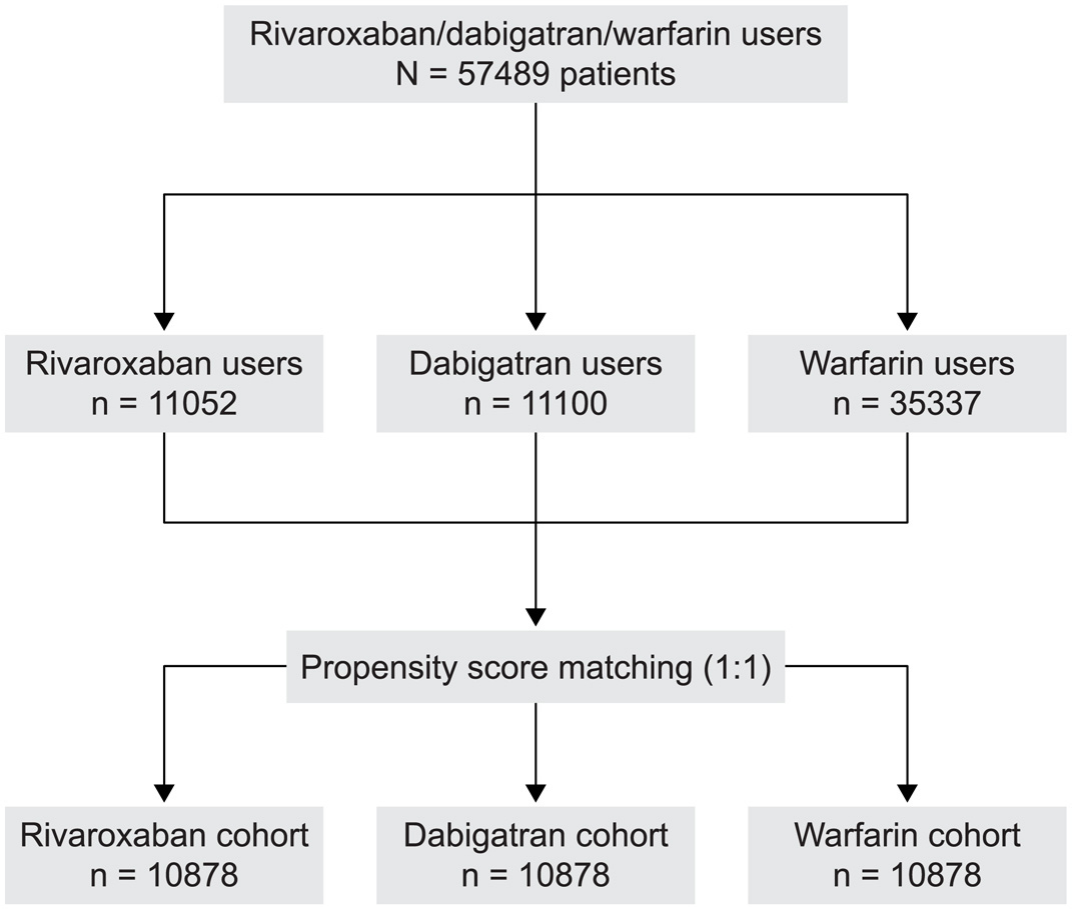
Patient flow diagram.

A total of 57489 patients (11052 receiving rivaroxaban, 11100 dabigatran, and 35337 warfarin) were eligible for inclusion in the analysis. After the two-phase propensity score-matching procedure, 32634 patients (10878 patients per OAC; Fig 1) were included. After matching, the baseline characteristics between the rivaroxaban, dabigatran, and warfarin cohorts were similar (Table 1). The mean lengths of follow-up were 329, 482, and 454 days for patients receiving rivaroxaban dabigatran, and warfarin, respectively.

**Table 1:** Baseline Patient Characteristics for the Propensity-Matched Population.

Characteristic	Rivaroxaban (R) Cohort (n = 10878)	Dabigatran (D) Cohort (n = 10878)	Warfarin (W) Cohort (n = 10878)	Standardized Difference W vs R, %	Standardized Difference W vs D, %	Standardized Difference D vs R, %
**Age, years, mean (SD)**	71.3 (11.1)	70.9 (10.8)	71.5 (11.3)	1.8	5.4	3.7
**Sex, male, %**	52.8	54.3	53.0	0.4	2.6	3.0
**Commercial payer (vs Medicare), %**	36.0	37.2	35.8	0.4	2.9	2.5
**Plan type, %**						
Comprehensive	36.1	35.1	36.2	0.2	2.3	2.1
EPO	0.4	0.4	0.3	1.7	1.7	0.0
HMO	9.4	9.1	9.2	0.7	0.4	0.1
POS	5.1	4.9	5.7	2.7	3.6	0.9
PPO	44.2	45.5	43.2	2.0	4.6	2.6
POS with capitation	0.2	0.3	0.4	3.7	1.7	2.0
CDHP	1.7	1.8	1.5	1.6	2.4	0.8
HDHP	0.7	0.7	0.9	2.3	2.3	0.0
**Geographic region, %**						
North East	18.1	18.9	18.2	0.3	1.8	2.1
North Central	28.4	30.1	26.7	3.8	7.6	3.7
South	36.6	34.1	37.4	1.7	6.9	5.2
West	15.3	15.3	16.2	2.5	2.5	0.0
Unknown	1.6	1.5	1.6	0.0	0.8	0.8
**CHA**_**2**_**DS**_**2**_**-VASc score, mean (SD)**	3.4 (1.30)	3.4 (1.32)	3.4 (1.26)	0.0	0.0	0.0
**CHADS**_**2**_ **score, mean (SD)**	1.85 (1.01)	1.84 (1.02)	1.85 (0.98)	0.0	1.0	1.0
**Heart failure, %**	18.7	19.3	18.6	0.3	1.8	1.5
**Hypertension, %**	73.5	71.8	73.9	0.9	4.7	3.8
**Age ≥75 years, %**	42.0	40.4	43.6	3.2	6.5	3.3
**Diabetes, %**	33.7	34.8	32.6	2.3	4.7	2.3
**Stroke/TIA, %**	8.7	8.9	8.2	1.8	2.5	0.7
**Vascular disease, %**	37.1	36.1	35.8	2.7	0.6	2.7
**Age 65–74 years, %**	29.6	30.4	28.2	3.1	4.8	1.8
**ATRIA risk score, mean (SD)**	2.10 (1.70)	2.06 (1.73)	2.17 (1.74)	4.1	6.3	2.3
**Anemia, %**	9.1	8.8	10.3	4.1	5.1	1.1
**Prior hemorrhage, %**	1.2	1.3	1.0	1.9	2.8	0.9
**HAS-BLED score, mean (SD)**	1.63 (0.70)	1.61 (0.72)	1.65 (0.71)	2.8	5.6	2.8
**Liver disease, %**	0.6	0.5	0.6	0.0	1.4	1.4
**Alcohol excess, %**	1.0	1.0	1.0	0.0	0.0	0.0
**Other comorbidities, %**						
**Myocardial infarction**	3.4	3.5	3.8	2.1	1.6	0.5
**Charlson comorbidity index score, mean (SD)**	1.24 (1.31)	1.25 (1.33)	1.19 (1.31)	3.8	4.5	0.8
0	33.8	33.8	35.4	3.4	3.4	0.0
1	34.0	34.1	35.4	2.9	2.7	0.2
2	17.7	17.2	16.6	2.9	1.6	1.3
3	8.0	8.2	7.2	3.0	3.8	0.7
4	3.9	3.9	3.6	1.6	1.6	0.0
5	1.7	1.5	1.6	0.8	0.8	1.6
≥6	1.0	1.3	1.1	1.0	1.8	2.8

ATRIA, Anticoagulation and risk factors in atrial fibrillation; CDHP, consumer-driven health plan; CHADS_2_, Congestive heart failure, Hypertension, Age ≥75 years, Diabetes mellitus, Stroke or transient ischemic attack (2 points); CHA_2_DS_2_-VASc, Congestive heart failure, Hypertension, Age ≥75 years (2 points), Diabetes mellitus, Stroke or transient ischemic attack (2 points), Vascular disease, Age 65–74, Sex category (female); EPO, exclusive provider organization; HAS-BLED, Hypertension, Abnormal renal/liver function, Stroke, Bleeding history or predisposition, Labile international normalized ratio, Elderly, Drugs/alcohol concomitantly; HDHP, high deductible health plan; HMO, health maintenance organization; POS, point of service; PPO, preferred provider organization; SD, standard deviation; TIA, transient ischemic attack

**Fig 2:**
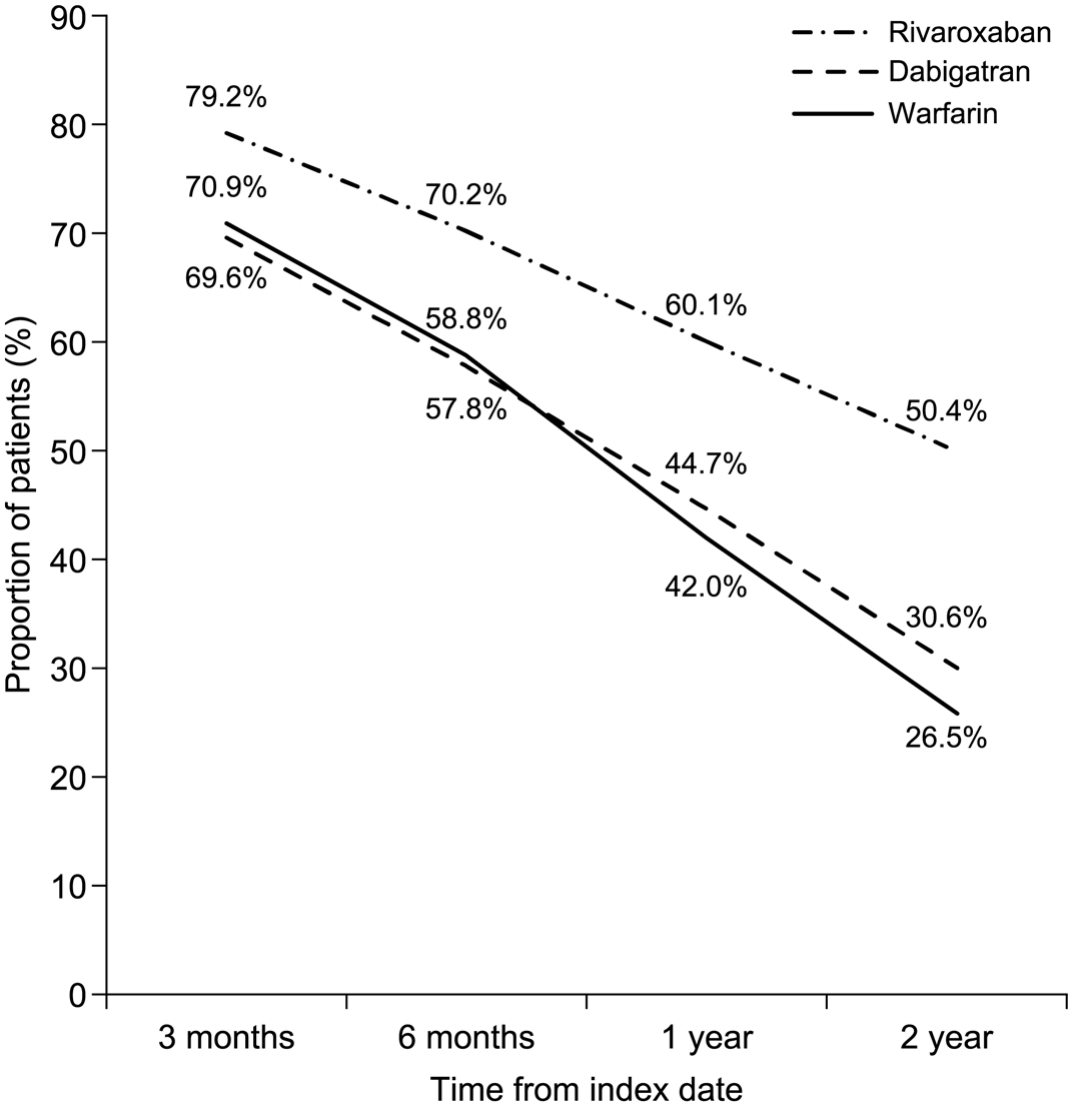
Treatment persistence according to oral anticoagulant therapy.

**Fig 3:**
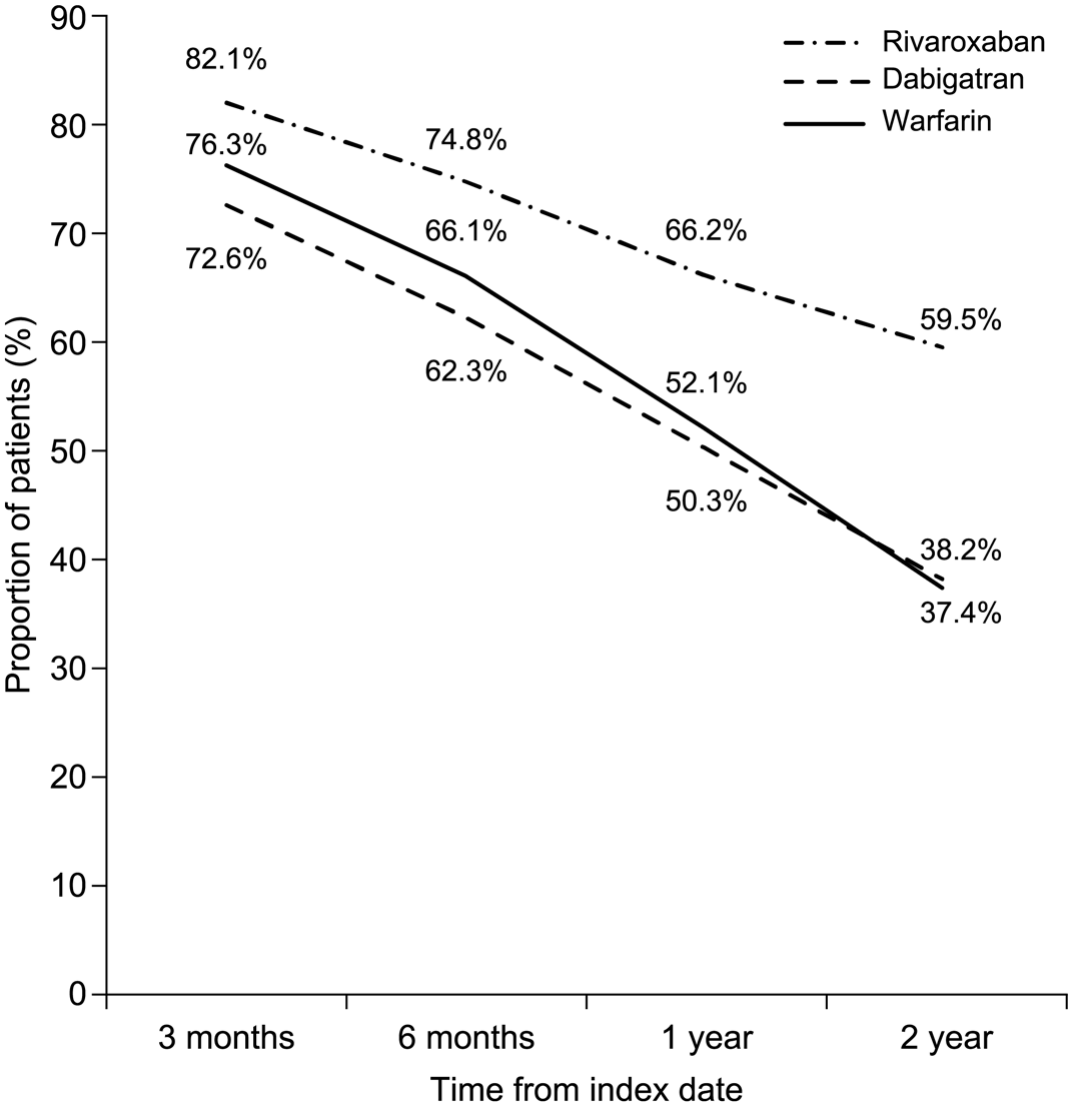
Treatment discontinuation according to oral anticoagulant therapy.

Kaplan–Meier curves for time to non-persistence are shown in Fig 2. After 3 months of follow-up, 79.2% of rivaroxaban users, 69.6% of dabigatran users, and 70.9% of warfarin users were persistent with treatment; these proportions decreased to 70.2%, 57.8%, and 58.8% by month 6; 60.1%, 44.7%, and 42.0%, respectively, by the end of year 1; and 50.4%, 30.6%, and 26.5%, respectively, by the end of year 2. On regression analysis, use of rivaroxaban was associated with significantly higher levels of persistence compared with dabigatran (HR 0.64, 95% CI 0.62–0.67) and warfarin (HR 0.62, 95% CI 0.59–0.64). Moreover, use of rivaroxaban was associated with a significantly lower rate of discontinuation than with dabigatran (HR 0.61, 95% CI 0.58–0.64) and warfarin (HR 0.65, 95% CI 0.62–0.68) (Fig 3). Dabigatran demonstrated significantly higher levels of persistence compared with warfarin (HR 1.05, 95% CI 1.01–1.10).

## Discussion

This is the first real-world US administrative claims database analysis to compare persistence and discontinuation rates of rivaroxaban, dabigatran, and warfarin directly in patients with NVAF. The OAC cohorts compared in this study were propensity score matched based on key baseline demographic and stroke and bleeding risk characteristics. The results demonstrated that patients receiving rivaroxaban had significantly higher persistence and lower discontinuation rates than those receiving either dabigatran or warfarin. Of note, however, non-persistence to therapy was high across all OACs, suggesting a failure of the medical community in stressing the important role of anticoagulants in preventing ischemic events due to NVAF.

Our findings are generally consistent with previous persistence/discontinuation studies performed using US claims databases [3–8]. Laliberté et al. [3] retrospectively analyzed administrative claims for 3654 rivaroxaban and 14616 warfarin users with NVAF between May 2011 and July 2012 in the Symphony Health Solutions’ Patient Transactional Dataset. After propensity score matching, the investigators found that rivaroxaban was associated with higher levels of persistence compared with warfarin over 180 days of follow-up (82.5% vs 69.8%; HR 0.66, 95% CI 0.60–0.72). These finding were echoed in a second propensity score-matched retrospective claims analysis of 7259 rivaroxaban and 7259 warfarin users identified in the Truven Health MarketScan Claims Databases between January 2009 and March 2013 [4]. Nelson et al. [4] identified significantly higher levels of persistence and lower discontinuation with rivaroxaban compared with warfarin over a mean follow-up of 184 days for rivaroxaban and 408 days for warfarin patients (64% vs 53%; adjusted HRs 0.63, 95% CI 0.59–0.68, p < 0.001, and 24% vs 48%; 0.54, 95% CI 0.49–0.58, p < 0.001, respectively). A retrospective analysis comparing therapy persistence with rivaroxaban and dabigatran, using administrative claims data for 7259 rivaroxaban users propensity score matched 1:1 with 7259 dabigatran users, suggested a higher likelihood of persistence at 1 year with rivaroxaban compared with dabigatran (64% vs 60%; adjusted HR 0.89, 95% CI 0.84–0.95) [8]. This comparative estimate of persistence was similar to that observed in our study. Considering the results of all these studies together [3,4,8,17], there appears to be substantial evidence to suggest that rivaroxaban is associated with increased persistence compared with either VKAs or dabigatran.

The HR for dabigatran versus warfarin persistence in our study suggests a smaller improvement with dabigatran (HR 0.95, p = 0.007) than seen in a previous study [7]. In their analysis of US Department of Defense administrative claims database, Zalesak et al. [7] found that patients who received dabigatran treatment had significantly longer median persistence than patients who received warfarin (>400 vs 222 days; p < 0.001 using a 60-day gap to define non-persistence). Persistence rates with warfarin were particularly low (e.g. 53.3% at 6 months) compared with rates observed in other recent US claims persistence studies [3,4], whereas the dabigatran persistence rate was relatively consistent with previous study estimates (71% at 6 months vs 58–70%) [7,8]. Another potential explanation for the better dabigatran versus warfarin persistence rates observed in the analysis by Zalesak et al. may be the timing of study performance. This study used data during the period in which dabigatran was first approved, and it is possible the higher persistence seen with dabigatran was bolstered by the initial absence of an alternative non-VKA OAC, which limited switching options for prescribers. Conversely, our current analysis began in November 2011, when both rivaroxaban and dabigatran were available.

Our finding that rivaroxaban therapy was associated with higher persistence and lower discontinuation than warfarin may be explained by the poor treatment satisfaction commonly reported by patients taking VKAs in a previous study [18]. Coleman et al. performed a cross-sectional survey study of patients with atrial fibrillation receiving thromboprophylaxis for stroke prevention, and found that warfarin users reported greater feelings of frustration and burden, resulting in a significant negative impact on patients’ lives compared with those receiving non-warfarin regimens (mostly acetylsalicylic acid alone) [18]. Their study further suggested that the frustration and burden associated with warfarin were the result of the dietary restrictions and the hassles of routine monitoring. Because rivaroxaban is a once daily medication, does not have many dietary interactions and does not require routine coagulation monitoring, patients are unlikely to have these burdens and, consequently, may remain on therapy longer.

The lower rates of persistence and higher rates of discontinuation reported for dabigatran compared with rivaroxaban may be a result of the frequent dyspepsia associated with dabigatran. The tartaric acid core formulation of dabigatran has been associated with a high incidence of gastrointestinal side-effects (~2-fold higher than with warfarin), with dyspepsia being among the most frequently reported adverse effects in those randomized to receive dabigatran in the Randomized Evaluation of Long-Term Anticoagulation Therapy (RE-LY) trial (11.3–11.8% in the dabigatran 110 mg and 150 mg arms) [19]. Although not formally tested in this analysis, it is possible that concerns around the potential of dabigatran to increase rates of myocardial ischemia/infarction risk [20], which were not shown in the corresponding rivaroxaban phase III Rivaroxaban Once daily, Oral, Direct Factor Xa Inhibition Compared with Vitamin K Antagonism for Prevention of Stroke and Embolism Trial in Atrial Fibrillation (ROCKET AF) study [21], may at least partially explain the lower rate of persistence and higher rate of discontinuation with dabigatran in our study. Further research is needed to better understand why patients are non-persistent with OAC therapy including the non-VKA OACs.

There are a number of limitations with this analysis that should be considered. MarketScan contains data on a subset of the US population and is not a random sample of patients. However, it is generally considered to be representative of the US population [11]. As with all administrative claims data, MarketScan data may contain billing code inaccuracies and missing data that can result in misclassification or other biases. The presence of residual confounding cannot be excluded in this analysis, because there may have been additional confounding factors not specified in MarketScan, which, therefore, could not be matched. The data analyzed were collected during the period immediately after rivaroxaban approval in the United States. In the intervening period, persistence patterns with rivaroxaban may have changed. The assessments of persistence and discontinuation were based on claims history that reflects only how medications were dispensed and not how they were taken. Finally, claims data cannot answer questions about the reasons for non-persistence or discontinuation.

In conclusion, the results of this retrospective administrative claims analysis demonstrate that patients with NVAF receiving anticoagulation for the prevention of stroke were more likely to persist with OAC therapy when prescribed rivaroxaban than either dabigatran or warfarin. Patients receiving rivaroxaban were also less likely to discontinue therapy. The introduction of novel OACs such as rivaroxaban have improved patient persistence to antithrombotic therapy, but much needs to be done to make patients with NVAF and their prescribers aware of the critical role of this treatment to reduce the thromboembolic risk in the long run. Future research to evaluate whether the relatively high persistence/low discontinuation rates for rivaroxaban translate into lower rates of ischemic stroke and does not produce a higher risk of bleeding in patients with NVAF is needed.
